# Ultrasound-Responsive Nrf2-Targeting siRNA-Loaded Nanobubbles for Enhancing the Treatment of Melanoma

**DOI:** 10.3390/pharmaceutics14020341

**Published:** 2022-01-31

**Authors:** Monica Argenziano, Federica Bessone, Chiara Dianzani, Marie Angèle Cucci, Margherita Grattarola, Stefania Pizzimenti, Roberta Cavalli

**Affiliations:** 1 Department of Drug Science and Technology, University of Turin, 10125 Turin, Italy; monica.argenziano@unito.it (M.A.); f.bessone@unito.it (F.B.); chiara.dianzani@unito.it (C.D.); 2 Department of Clinical and Biological Science, University of Turin, 10125 Turin, Italy; marieangele.cucci@unito.it (M.A.C.); margherita.grattarola@unito.it (M.G.); stefania.pizzimenti@unito.it (S.P.)

**Keywords:** nanobubbles, ultrasound, small interfering RNA (siRNA), nuclear factor erythroid 2-related factor 2 (Nrf2), melanoma, drug resistance

## Abstract

The siRNA-mediated inhibition of nuclear factor E2-related factor 2 (Nrf2) can be an attractive approach to overcome chemoresistance in various malignant tumors, including melanoma. This work aims at designing a new type of chitosan-shelled nanobubble for the delivery of siRNA against Nrf2 in combination with an ultrasound. A new preparation method based on a water–oil–water (W/O/W) double-emulsion was purposely developed for siRNA encapsulation in aqueous droplets within a nanobubble core. Stable, very small NB formulations were obtained, with sizes of about 100 nm and a positive surface charge. siRNA was efficiently loaded in NBs, reaching an encapsulation efficiency of about 90%. siNrf2-NBs downregulated the target gene in M14 cells, sensitizing the resistant melanoma cells to the cisplatin treatment. The combination with US favored NB cell uptake and transfection efficiency. Based on the results, nanobubbles have shown to be a promising US responsive tool for siRNA delivery, able to overcome chemoresistance in melanoma cancer cells.

## 1. Introduction

While it still accounts for less than 5% of all cutaneous malignancies, melanoma is the most lethal form of skin cancer [1]. Metastatic melanoma (MM) is poorly responsive to treatment based on conventional chemotherapy, resulting in a 5-year survival rate of only 15% [2]. Over the past few years, new targeted treatments and immunotherapy [3,4] have significantly improved the global approach toward melanoma. Activating mutations in cytoplasmic serine/threonine kinase B-Raf (BRAF), belonging to the mitogen-activated protein kinase (MAPK) signaling pathway, are the most frequent genetic alterations present in approximately 50% of all melanoma cases [5]. Mutated BRAF elicits a constitute activation of the MAPK signaling pathway. Thus, BRAF inhibitors (BRAFi), such as vemurafenib or dabrafenib, together with inhibitors of the mitogen-activated protein kinase (MEK) (i.e., trametinib), a BRAF downstream effector, have been successfully employed in patients with advanced mutated BRAF melanoma diseases [6]. For BRAF wild-type MM patients, the current guidelines recommend the use of monoclonal antibodies targeting immune checkpoint proteins, such the anti-programmed death 1 (PD-1) (pembrolizumab or nivolumab) or the cytotoxic T-lymphocyte antigen 4 (CTLA-4) (ipilimumab), in combination with an anti-PD-1 therapy [7]. These new agents improved survival compared with chemotherapy alone. However, a significant number of patients exhibit intrinsic resistance or develop it during treatment with these modern therapies, determining a real limit to their efficacy [8,9]. Understanding these mechanisms is one of the mainstreams of the research in the successful treatment of MM. 

Several genetic and epigenetic mechanisms have been described in MM resistance to therapeutical approaches [8,9]. However, in recent years, researchers have focused their attention on oxidative stress being able to play a central role in tumor progression [10], including melanoma [11]. Indeed, a growing body of evidence indicates that, compared with normal, healthy tissue, tumor tissues exhibit a high level of reactive oxygen species (ROS), which allow the activation of the pro-tumorigenic signaling pathway [10]. To maintain acceptable sublethal ROS levels, cancer cells usually increase their antioxidant systems to protect cells from oxidative stress damage and favor their survival. However, this adaptative process seems to elicit the rise of more resistant cell subclones [12]. In particular, nuclear factor E2-related factor 2 (Nrf2) [13], a transcription factor able to induce the expression of several antioxidant and cytoprotective genes, seems to play a key role in the progression of melanoma [10], as well as in several other types of cancer [14]. In low oxidative stress conditions, Nrf2 is found in the cytosol linked to its inhibitor, Keap1 (Kelch-like ECH-associated protein), which facilitates Nrf2 ubiquitination and proteasomal degradation. Under oxidative stress, Keap1 undergoes a conformational change due to the oxidation of its cysteine residues and releases Nrf2. Therefore, this transcription factor can translocate into the nucleus and bind to the antioxidant response element (ARE) sequences present in the promoter of genes coding for several antioxidant enzymes, such as Heme oxygenase-1 (HO-1) and glutathione-S-transferase (GST), as well as the genes involved in GSH synthesis, such as γ-glutamate-cysteine ligase (GCL), which catalyzes the first step in the production of GSH [13]. Scientists initially attribute a protective role for Nrf2 at the onset of malignant transformation, related to its carcinogen-detoxifying ability and protective action toward oxidative stress.

However, accumulating evidence shows an opposite role during tumor progression, where the upregulation of Nrf2 has been observed to affect cell proliferation, epithelial-mesenchymal transition (EMT), migration, invasion, and angiogenesis [15], as well as in the chemo- and radioresistance of various malignant tumors, including melanoma [16,17,18]. For instance, IHC studies on malignant melanoma samples have revealed that Nrf2 expression was correlated with deeper Breslow, an invasive phenotype, nodular growth, and worse survival [19]. Moreover, several data showed an aberrant activation of Nrf2 in chemoresistant melanoma cells and demonstrated that its downregulation, also through the small interfering RNA (siRNA) approach, enhanced melanoma sensitivity to conventional drugs [20,21]. Interestingly, Nrf2 activation seems to be also involved in an acquired resistance to molecularly targeted therapy, such as anti-BRAF treatment [22], and Nrf2 inhibition can overcome melanoma radioresistance [18]. For these reasons, Nrf2 can be a promising target to predict tumor chemosensitivity, and its inhibition can overcome chemo- and radioresistance.

Several pharmacological strategies have been proposed to inhibit Nrf2 to overcome chemoresistance. Among them, Brusatol, a quassinoid from the plant *Brucea javanica*, has increasingly gained attention [23,24]. More recently, we demonstrated that another quassinoid, Ailanthone, obtained from the Ailanthus altissima plant, is a potent inhibitor of Nrf2, able to overcome the chemoresistance to cisplatin in bladder and ovarian cancer [25,26]. However, their mechanisms of action can be nonspecific. An enhanced specificity can be achieved by using a specific siRNA against the Nrf2 gene. siRNAs are small double-stranded RNA molecules, typically 21–24 nucleotides in length, with two nucleotide 3′-overhangs, which consist of a guide strand (antisense) and passenger strand (sense). Notably, the siRNA tool has been extensively applied to inhibit tumor-promoting factors and reverse chemoresistance [27,28].

However, siRNA clinical application is still limited, because siRNA is prone to quick enzymatic degradation in systemic circulation, rapid renal clearance, and poor cell uptake due to its hydrophilicity, high molecular weight, and negative charge. Moreover, it needs to overcome several biological barriers to reach the cytoplasm of target cells [29,30].

The nanotechnology-based therapeutic approach represents a promising strategy to overcome these limitations and to improve the performance of siRNA concerning the stability, specificity, and potential off-target effects [31]. Indeed, nanocarriers can extend the siRNA circulation time in the bloodstream, avoiding nuclease degradation. Moreover, they can favor cell uptake and deliver siRNA to precise tissues by active targeting or exploiting nanocarrier preferential accumulation in the tumor site by passive targeting thanks to the Enhanced Permeability and Retention (EPR) effect [32,33]. Alternatively, external stimuli (i.e., ultrasound and magnetic field) can be applied to trigger siRNA release to a specific site [34]. A large number of nanoformulations, including liposomes, lipid nanoparticles, polymeric nanoparticles, dendrimers, and inorganic nanoparticles, has been proposed for siRNA delivery [33,34,35]. In this field, Patisiran (ONPATTRO^®^) was the first siRNA nanoformulation approved by the Food and Drug Administration (FDA) in 2018 for the treatment of hereditary transthyretin-mediated amyloidosis [36]. The nanosystem is based on lipid nanoparticles that encapsulate a chemically modified siRNA [31]. The clinical success of effective siRNA delivery with a nanoformulation has paved the way for the exploration of other nanocarrier technologies for siRNA administration. In this context, polymer-shelled nanobubbles (NBs) have shown great potential as a nanoplatform for efficient nucleic acid delivery [37,38,39,40,41]. Interestingly, they can be combined with an ultrasound (US) to improve the delivery of active molecules [42]. Therefore, US-triggered gene delivery with NBs can be advantageous, since the release of their payload can be obtained in specific sites in response to US application [43,44,45]. This property might be usefully proposed to treat the cutaneous metastasis of melanoma, considering that US are currently in clinical practice. 

The aim of the work was the design of a new type of chitosan-shelled nanobubble for the delivery of siRNA against Nrf2 in combination with US. The NB formulations were conceived, prepared, and in vitro characterized from the physicochemical point of view. Moreover, the biological activity of siNrf2-NBs in improving cisplatin sensitivity in a drug resistance melanoma model was evaluated. 

## 2. Materials and Methods

### 2.1. Reagents

Unless otherwise stated, all the reagents were from Sigma Aldrich (St. Louis, MO, USA). Soybean lecithin (Epikuron 200^®^) was from Cargill (Hamburg, Germany). Chitosan low molecular weight (degree of deacetylation 75–85%, 50–190 KDa) was used.

### 2.2. Preparation of siNrf2-Nanobubble Formulations

Blank and siRNA-loaded NBs (siNrf2-NBs) were prepared developing a new preparation method based on a water–oil–water (W/O/W) double-emulsion. Briefly, 55 µL of anti-Nrf2 siRNA (SI03246950, Qiagen S.R.L., Milan, Italy and 5′-CAUUUGAUGUUUCUGAUCUATT-3′) aqueous solution (100 µM) containing Tween80^®^ (1.5% *w*/*v*) were added to 195 µL of decafluoropentane. The mixture was sonicated using a 220–230 V Bransonic^®^ 3510 Ultrasonic Cleaner (Emerson; St. Louis, MO, USA) in an ice bath for 2 min, producing the first w/o emulsion. Then, the w/o emulsion was dropwise added to 3 mL of a 5.5% *w*/*v* glucose aqueous solution containing soybean lecithin (Epikuron^®^, 3% *w*/*v*). The system was sonicated using an Ultrasonic Generator (20 K, 500 W; Hainrtec, type HNG-20500-SP) for 1 min in an ice bath. Finally, to obtain the chitosan-shelled NBs, an aqueous solution of chitosan (150 µL, 2% *w*/*v*, pH 5.0) was dropwise added to the preformed NBs under gentle magnetic stirring. NBs loaded with a control-negative siRNA (Eurofins Genomics Germany GmbH, Ebersberg, Germany) were also prepared (siRNAneg-NBs). Blank chitosan-shelled NBs were formulated in the absence of siNrf2 using the same procedure. Fluorescent NBs were obtained adding 6-coumarin to the decafluoropentane (0.1% *w*/*v*).

### 2.3. Characterization of siNrf2-Nanobubble Formulations

Blank (NBs), siNrf2-NBs, siRNAneg-NBs, and fluorescent 6-coumarin-loaded NBs were in vitro characterized measuring the physicochemical parameters (i.e., average diameter, polydispersity index, and zeta potential) by Dynamic Light Scattering (DLS). A 90 Plus Instrument (Brookhaven, New York City, NY, USA) was used. The analyses were performed at a scattering angle of 90° and at 25 °C on NB samples diluted in water (1:30 *v*/*v*). The zeta potential values were determined by placing diluted NB samples in an electrophoretic cell, where an approximately 15-V/cm electric field was applied. All measurements were conducted in triplicate. The pH and the osmolarity of the samples were determined at room temperature using pHmeter Orion (model 420A, from Thermo Scientific, Waltham, MA, USA) and Semi-Micro Osmometer K-7400 Knauer (Berlin, Germany), respectively.

Transmission electron microscopy (TEM) analysis was performed to evaluate NB morphology. A Philips CM10 (Eindhoven, The Netherlands) instrument was used. The diluted NB aqueous suspensions were sprayed on a Formwar-coated copper grid and air-dried before observation.

The gel retardation assay using electrophoresis on agarose gel was carried out to confirm the incorporation of the anti-Nrf2 siRNA within the NBs. The samples were stained with an ethidium bromide solution (0.5 µg/mL) and were loaded onto the agarose gel (2% *w/v*). Free siRNA and unloaded NBs were used as positive and negative controls, respectively. The electrophoresis was run in TAE buffer (40-mM Tris base, 20-mM acetic acid, and 1-mM EDTA; pH 8.0) at 100 V for 30 min. The banding pattern was visualized using an ultraviolet transilluminator and photographed with a Polaroid camera (Kodak, Rochester, NY, USA).

Polyacrylamide gel analysis was performed using 13% acrylamide gels loaded with 20 µL of siNrf2-NB samples and 6 µL of loading buffer containing 25% glycerol and 0.2% bromophenol blue (Thermo Fisher Scientific, Waltham, MA, USA). Electrophoresis was carried out at a voltage of 100 V for 90 min in TAE buffer. The gels were then stained with ethidium bromide solution (0.5 µg/mL) and visualized using an ultraviolet transilluminator equipped with a Polaroid camera.

The encapsulation efficiency of siNrf2-NBs was measured from the quantification of free siRNA after NB centrifugation (15,000 rpm, 15 min, 4 °C) using an Amicon^®^ Ultra-0.5 centrifugal filter unit (Sigma Aldrich, St. Louis, MO, USA). The concentration of free siRNA in the filtrate was determined by spectrophotometric analysis using an UV–visible spectrophotometer (VICTOR X; Multilplate Reader, Perkin Elmer, Waltham, MA, USA) set at the wavelength of 260 nm. The encapsulation efficiency (%) was the percentage of loaded siRNA calculated as the difference between the total amount of siRNA used for the NB preparation and the amount of free siRNA to the total amount of siRNA added.

### 2.4. In Vitro Release Studies

The in vitro release of anti-Nrf2 siRNA from the NBs was evaluated in phosphate-buffered saline (PBS) 0.05 M at pH 7.4. The siNrf2-NBs were incubated with the receiving medium at a 1:10 *v*/*v* ratio under magnetic stirring over time. At fixed times, an aliquot was withdrawn, and the same volume of fresh PBS was added. Centrifugal filtration (Amicon^®^ Ultra MW cut-off 30 kDa, Sigma-Aldrich, St. Louis, MO, USA) was used to separate the released siRNA from the siNrf2-NBs. The released siRNA concentration in the filtrate was measured by a spectrophotometric analysis (wavelength of 260 nm, VICTOR X; Multilplate Reader). Moreover, the gel retardation assay was performed on the withdrawn samples, as previously described. 

### 2.5. In Vitro Stability Studies

The physical stability of the NB formulations stored at 4 °C was evaluated over time, determining the average diameter, Z-potential, and morphology of the samples up to 6 months. Moreover, the gel retardation assay, using electrophoresis in an agarose gel, was performed to confirm the siNrf2 incorporation within the NBs over time. 

### 2.6. Evaluation of Nanobubble Haemolytic Activity

The hemolytic activity of the NB samples was determined using rat blood diluted with phosphate-buffered saline (PBS) at pH 7.4 (1:10 *v*/*v*). A series of PBS dilutions of each sample (1:10, 1:25, 1:50, 1:100, 1:250, and 1:500 *v/v*) was incubated with the diluted blood at 37 °C for 90 min. The samples were then centrifuged (2000 rpm, 10 min), and the amount of hemoglobin released in the supernatant due to hemolysis was quantified by a spectrophotometric analysis at 543 nm (Du 730 spectrophotometer; Beckman Coulter, Fullerton, CA, USA).

The hemolytic activity was calculated with reference to the positive control, which was a complete hemolyzed blood sample due to a Triton X-100 (1% *w*/*v*) addition, and the negative control (NaCl 0.9% *w*/*v*).

### 2.7. Cell Line

M14 melanoma cells were kindly provided by Dr. Pistoia (Gaslini Institute, Genoa, Italy); cells were cultured in RPMI 1640 and supplemented with 10% fetal bovine serum (FBS), 100 units/mL of penicillin, and 100 μg/mL of streptomycin (Euroclone, Pero, Milan, Italy) in a 5% CO_2_ 37 °C incubator.

### 2.8. Evaluation of Nanobubble Cellular Internalization in M14 Cells under Fluorescence Miscroscopy

NB internalization was determined by using fluorescent 6-coumarin-NBs. M14 cells (5000 cells/wells) were plated into the channels of a µ–Slide VI0.4 (Ibidi, Giemme Snc, Milano, Italy) for 24 h to achieve approximately 70% confluence. Internalization of 6-coumarin-NBs was analyzed by fluorescence microscopy (Axiovert 35, Zeiss, Oberkochen, Germany).

### 2.9. siNrf2 Transfection with a Traditional Protocol

The effects of the siNrf2-NB treatment in M14 cells were compared with those obtained by exposing cells to the same amount of naked siNrf2 (SI03246950, Qiagen, Milan, Italy) transfected with the HiPerFect^®^ Transfection Reagent (301705, Qiagen, Milan, Italy) with the traditional protocols suggested by the manufacturer and as previously reported [35].

### 2.10. Western Blot

β-actin (#4970S, Cell Signaling Technology, Danvers, MA, USA) and Nrf2 (sc-722, Santa Cruz Biotechnology, Heidelberg, Germany) antibodies were used for the Western bot analysis, which was carried out as previously reported [35,46].

### 2.11. Viability Analysis in M14 Cell Line after siNrf2-Nanobubble Treatment

M14 (1500 cells/wells) were seeded in a 96-well plate with 100 μL of serum-supplemented medium. After cell treatments, the viability was assessed by the MTT (3-(4,5-dimethyl thiazol-2-yl)-2,5-diphenyltetrazolium bromide) (Merck Life Science S.R.L., Roma, Italy) assay, as previously reported [25].

### 2.12. Viability Analysis in M14 Cell Line after Ultrasound Irradiation

M14 were transferred in separated tubes (4000 cells/tube) in 200 μL of serum-supplemented medium. Cells were insonated for 0, 5, 10, 15, 30, and 60 s by using an ultrasound probe with an oscillation frequency of 2.5 ± 0.1 MHz. As soon as after insonation, cells were transferred in a 96-well plate in a 37 °C incubator. After 24 h, viability was assessed by the MTT analysis as described in Section 2.11. 

### 2.13. Cytofluorimetric Evaluation of NB Cellular Internalizazion in M14 Cells after Ultrasound Irradiation

M14 were transferred in separate tubes (4000 cells/tube) in 200 μL of serum-supplemented medium. Fluorescent 6-coumarin-NBs were added to M14 cells, and US was applied for 10 s, as described above. Cells were then incubated for 5, 15, or 30 min in a 37 °C incubator. After incubation, cells were centrifugated at 1000 rpm for 10 min at 4 °C; the pellets were collected and resuspended in 500 μL of PBS1x. Internalization of 6-coumarin-NBs was evaluated by using a FACScan cytometer (Becton Dickinson, Accuri, Eysins, Vaud. Switzerland). Results were compared to those obtained in nonsonified M14 cells in the same experimental setting. 

### 2.14. Transfection Efficiency of siNrf2-NB M14 Cells after Ultrasound Irradiation

M14 were transferred in separated tubes (350,000 cells/tube) in 2 mL of serum-supplemented medium. Cells were incubated with 0.08-µM siNrf2-NB or 0.08-µM siRNAneg-NB, and 10 s of ultrasound irradiation was applied. Thereafter, cells were seeded into 6-well tissue culture plates and incubated for 24 h and 48 h in a 37 °C incubator. At the indicated times, cells were collected for WB analysis of the Nrf2 protein expression, as described in Section 2.10.

### 2.15. Echogenic Properties of NB Formulations

An aqueous suspension of siNrf2-NBs at a concentration of 1·10^12^ NBs/mL was added into a tank of ultrapure water and kept under magnetic stirring. To reduce the acoustic reflections, an acoustic absorbing pad was placed at the tank’s bottom. The NBs were insonified using a US clinical echomachine (MyLab™ 25Gold; Esaote, Genova, Italy) operating in B mode. B mode cineloops were acquired at increasing mechanical indices (MI). B mode cineloops of water in the absence of NBs were also acquired as the control.

### 2.16. Statistical Analysis

With GraphPad InStat software (San Diego, CA, USA), we performed a one-way ANOVA analysis followed by the Bonferroni multiple comparison post-test to evaluate the differences between experimental groups. Values of *p* ≤ 0.05 were considered statistically significant.

## 3. Results and Discussion

### 3.1. Characterization of siNrf2-Nanobubble Formulations

The rationale of the work was the design and development of a suitable US-sensitive nanocarrier for the effective intracellular delivery of siRNA to overcome cisplatin resistance. The main parameters necessary for protecting siRNA from enzymatic degradation, overcoming biological barriers and enabling siRNA to be released intracellularly, were taken into account for the formulation design. Indeed, it is worth noting that the nanodelivery system physicochemical characteristics such as size, surface charge, and morphology strongly affect the nanoparticle biological fate, including blood circulation and uptake by cancer cells [32,47].

**Figure 1:**
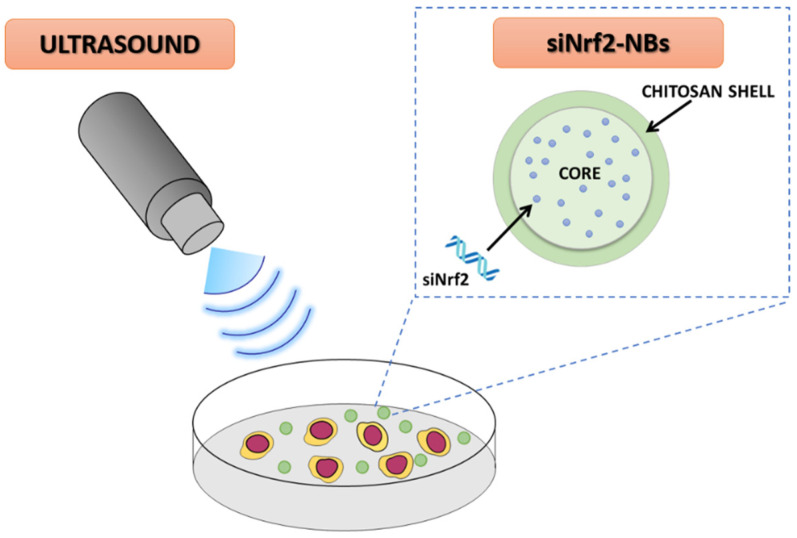
Schematic representation of the ultrasound-responsive Nrf2-targeting siRNA-loaded nanobubbles (siNrf2-NBs).

Based on these premises, a purposely tailored nanobubble architecture was developed (Figure 1). In this nanostructure, siRNA was encapsulated in aqueous nanodroplets inside the decafluoropentane core of chitosan-shelled nanobubbles (NBs).

The formulation herein reported is referred to as “nanobubbles” for the sake of simplicity, but it would be more accurate to use the term “nanovesicles” when the core constitutes decafluoropentane, a perfluorocarbon liquid at room temperature. However, the nanosystem has a vaporizable core, since it can be activated by US, which causes a decafluoropentane liquid-to-vapor transition via the acoustic droplet vaporization (ADV) phenomenon, leading to the change from droplet to bubbles. The chitosan-shelled NBs were obtained exploiting water-oil-water (W/O/W) double-emulsion as a template. The nanoemulsion preparation protocol was purposely tuned for the loading of siRNA, employing mild conditions and no organic solvents to assure the siRNA stability. NBs loaded with anti-Nrf2 siRNA (siNrf2-NBs) or with a negative control siRNA (siRNAneg-NBs) were prepared, dissolving siRNA in the internal water phase of the W/O/W emulsion. The core localization was conceived to assure nucleic acid protection from the external environment and to increase the payload. Fluorescent chitosan-shelled NBs were then prepared by adding 6-coumarin in the decafluoropentane core. Table 1 reports the physicochemical characteristics of the fluorescent and siRNA-loaded NBs, as well as the blank formulations.

The preparation method allowed the production of a very small siRNA-loaded nanoplatform. All the NB formulations showed sizes of about 100 nm and a narrow size distribution (PDI of about 0.2), suitable parameters for potential i.v. administration. Moreover, the very small sizes of NBs can play an important role in cell uptake. Cellular internalization markedly depends on the nanocarrier size, as well as the charge and morphology [48]. Additionally, the combination of US with NBs can improve siRNA cell accumulation due to the permeabilization of the cell membrane [44].

**Figure 2:**
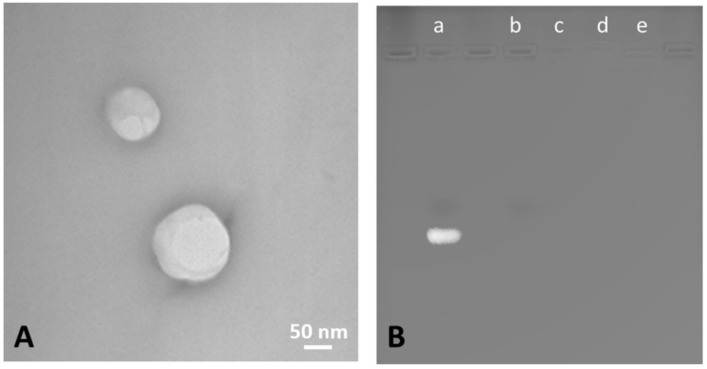
(**A**) TEM image of siNrf2-NBs (magnification 39,000×, scale bar 50 nm). (**B**) The gel retardation assay using electrophoresis on agarose gel of (a) naked siRNA, (b) blank NBs, (c) siNrf2-NBs, (d) siNrf2-NBs stored at 4 °C for 6 months, and (e) siNrf2-NBs after 6 h of incubation in PBS, pH 7.4.

The NBs had a spherical morphology and a well-defined core–shell structure. A representative TEM image of the siNrf2-NBs is shown in Figure 2A. Moreover, the TEM analysis confirmed the sizes of the NBs measured by DLS. 

All the NB formulations showed a positive surface charge, with a zeta potential of about +26 mV, a value high enough to avoid nanoparticle agglomeration/aggregation. The positive charge is related to the presence of a positively charged chitosan shell on the NB surface.

The stable chitosan NB coating was achieved by exploiting the capability of the cationic polysaccharide to interact with the interfacial phospholipid monolayer of the NB system [49]. Indeed, chitosan–phosphatidylcholine electrostatic and hydrophobic interactions have been previously described in the literature [50,51]. 

Previously, chitosan was widely investigated for siRNA delivery due to the favorable properties such as a positive charge, low toxicity, low immunogenicity, biocompatibility, and biodegradability [52,53,54]. Interestingly, much research has been focused on siRNA complexation with chitosan into nanoparticles/polyplexes [55].

In addition, siRNA complexation with cationic polymers via electrostatic interactions has been largely studied [56,57,58]. However, this approach suffers from some limitations, such as the stability issue in biological fluids, low transfection efficiency, and toxicity problems [45,55,59]. The formulation method herein reported entails siRNA incorporation with a different strategy. siRNA was encapsulated within the NB core, and the loading was not related to electrostatic interactions with the chitosan-positive amino groups present in the shell. Indeed, no significant difference in the size or zeta potential values was observed between the blank and siRNA-loaded NBs (Table 1). The absence of a size decrease showed that no condensation of the polysaccharide chains in the NB shell after siRNA incorporation occurred. This behavior confirmed that siRNA is encapsulated in nanoreservoirs inside the NB perfluorocarbon core and not adsorbed on the positively charged polymer shell. This loading strategy here reported was designed to increase the payload and provide additional protection from siRNA degradation in blood circulation. An encapsulation efficiency of 90.12 ± 0.38% was reached for siNrf2-NBs, with a siRNA concentration of about 50 µg/mL.

The incorporation of siRNA within the NBs was then confirmed by the gel retardation assay using electrophoresis on agarose gel (Figure 2B). Indeed, siRNA loaded in NBs was completely prevented from migrating through the gel, indicating its incorporation within the NB structure. On the contrary, a marked band was clearly detected for naked siRNA.

Moreover, polyacrylamide gel analysis was performed to detect unencapsulated siRNA with more sensitivity. Only naked siRNA was observed as a bright band, whereas the encapsulation in NBs prevented siRNA migration through the polyacrylamide gel. As far as stability issues, the NBs exhibited no significant changes in the average diameter, polydispersity index, and zeta potential values for up to 6 months, indicating good physical stability of the formulations during storage at 4 °C. The NB zeta potential values (about +26 mV) were high enough for all the formulations to assure the colloidal system physical stability over time and prevent aggregation phenomena thanks to high electrostatic repulsion between positive-charged nanoparticles. In addition, the in vitro stability over time of the siNrf2-NBs stored at 4 °C was also checked by agarose gel electrophoresis. The complete retardation of siRNA mobility in the NBs was achieved even after 6 months. 

The in vitro release kinetics of the siRNA from NBs was investigated in PBS at pH 7.4. After 6 h of incubation, no bands were observed in the agarose gel, indicating that siRNA was retained in the NB nanostructure. The siRNA might be released from NBs by diffusion through the chitosan shell, and the release kinetics are not fully dependent on the degree of protonation of chitosan amino groups.

No hemolytic activity was observed for all the NB samples, suggesting the biocompatibility of the formulations.

### 3.2. Evaluation of NB Cellular Internalization

**Figure 3:**
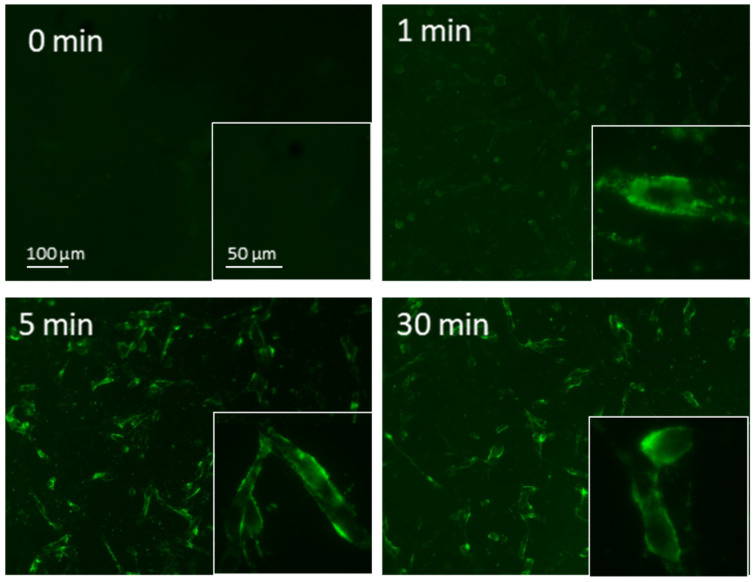
Fluorescent images of 6-coumarin-NB uptake in M14 cells at the indicated times. Green fluorescence of 6-coumarin was examined by using fluorescence microscopy (454 nm).

The cellular internalization of NBs in M14 cells was evaluated by fluorescence microscopy using fluorescent-labeled 6-coumarin-NBs. Since siRNA is unable to cross cellular membranes due to its hydrophilic and anionic nature, its encapsulation in NBs might increase the cellular uptake [60]. As shown in Figure 3, the fluorescent-labeled 6-coumarin-NBs were internalized within 1 min into the M14 cell line, and after 5 min of incubation, its accumulation significantly increased, lasting for 30 min. 

The fast internalization of chitosan NB formulations by the cells has been already reported in previous research [61,62,63]. This extremely rapid entry of NBs into cells strongly supports the possibility of effective drug delivery.

### 3.3. Biological Evaluation of siNrf2-NB on M14 Human Melanoma Cell Line

**Figure 4:**
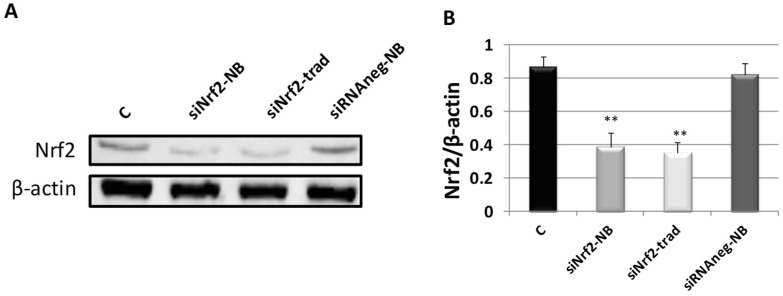
(**A**)Western blot analysis of Nrf2 in M14 untreated cells (C, control), treated with 0.08-µM siNrf2-NB (siNrf2-NB) or 0.08-µM negative control siRNA (siRNAneg-NB), or transfected with the same amount of siNrf2 (siNrf2-trad) in HiPerFect^®^ reagent with the traditional method at 24 h. (**B**) Densitometric values of Nrf2 blot normalized using the β-actin signal. Data are the mean ± SD from three independent experiments. ** *p* ≤ 0.01 vs. C.

M14 human melanoma cells were treated with siNrf2-NB. The in vitro transfection efficiency and specificity of siNrf2-NB were determined by Western blot (WB) for Nrf2 at 24 h (sc 722, Santa Cruz, CA, USA). The results were compared with those obtained after transfecting cells with the same amount of siNrf2 in HiPerFect^®^ reagent with the traditional method (siNrf2-trad) or negative control siRNA loaded in NB (siRNAneg-NB) (Figure 4). The Nrf2 protein content was downregulated at 24 h after the siNrf2-NB treatment, similar to the obtained siNrf2 transfection with the traditional method. The treatment with siRNAneg-NB did not inhibit Nrf2 expression, which remained at similar levels compared to the control. 

**Figure 5:**
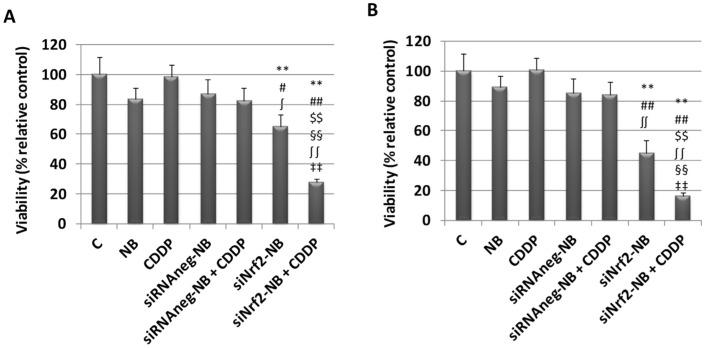
Viability was determined by the MTT assay at 24 h (**A**) and 48 h (**B**) in M14 untreated cells (C, control) or treated with blank NB, 1 µg/mL CDDP, and 0.08 µM siRNAneg-NB or siRNAneg-NB in combination with CDDP (siRNAneg-NB+CDDP) and 0.08 µM siNrf2-NB or siNrf2-NB in combination with CDDP (siNrf2-NB+CDDP). Results are expressed as the percent of the relative control values and are the mean ± standard deviation of three separate experiments performed in triplicate. ** *p* ≤ 0.01 vs. C; # *p* ≤ 0.05 and ## *p* ≤ 0.01 vs. NB; $$ *p* ≤ 0.01 vs. CDDP; ʃ *p* ≤ 0.05 and ʃʃ *p* ≤ 0.01 vs. siRNAneg-NB; §§ *p* ≤ 0.01 vs. siNrf2-NB; and ‡‡ *p* ≤ 0.01 vs. siRNAneg-NB + CDDP.

The ability of siNrf2-NB in reducing drug resistance was checked in melanoma M14 cells by analyzing the cytotoxicity (MTT test) after single and combined treatments with siNrf2-NB and cis-diammineplatinum(II) dichloride (cisplatin or CDDP). As shown in Figure 5, we observed a significant downregulation on the viability in cells treated with 0.08-µM siNrf2-NB at 24 h and 48 h. A greater inhibition was observed in cells treated with both 0.08-µM siNrf2 and 1-µg/mL CDDP (siNrf2-NB+CDDP) at 24 h and 48 h, demonstrating that the inhibition of Nrf2 sensitized melanoma cells to the CDDP treatment. On the contrary, the treatments with blank NB, 1 µg/mL CDDP, siRNAneg-NB, or siRNAneg-NB+CDDP were ineffective at 24 h and 48 h. The inhibition after the combined treatment was significant not only in comparison to the control values but also to those obtained after the blank NB, CDDP, siNrf2-NB, siRNAneg-NB, or siRNAneg-NB+CDDP treatments at 24 h and 48 h.

Worthy of note is the increasing attention paid to the use of nanocarriers for siRNA delivery to reverse CDDP resistance, as shown by the large number of studies found in the literature [64]. Among them, phospholipid NBs loaded with long intergenic noncoding RNA 00511-small interfering RNA (LINC00511-siRNA) were recently investigated by Wu et al. [41,65] for suppressing CDDP resistance in triple-negative breast cancer.

### 3.4. Ultrasound-Mediated siNrf2-NB Delivery in M14 Cell Line

It has been shown that the use of US as an external stimulus is a promising physical method to enhance transfection efficiency [44]. Ultrasound-mediated siRNA delivery based on nanobubbles has recently gained increasing attention due to its great potential to locally improve gene delivery and cell internalization, obtaining site-specific effects [66]. To verify this effect in our experimental model, both the NB cellular uptake and siNrf2-NB transfection efficiency in M14 cells after US exposure were evaluated and compared with the results obtained in non-sonified M14.

**Figure 6:**
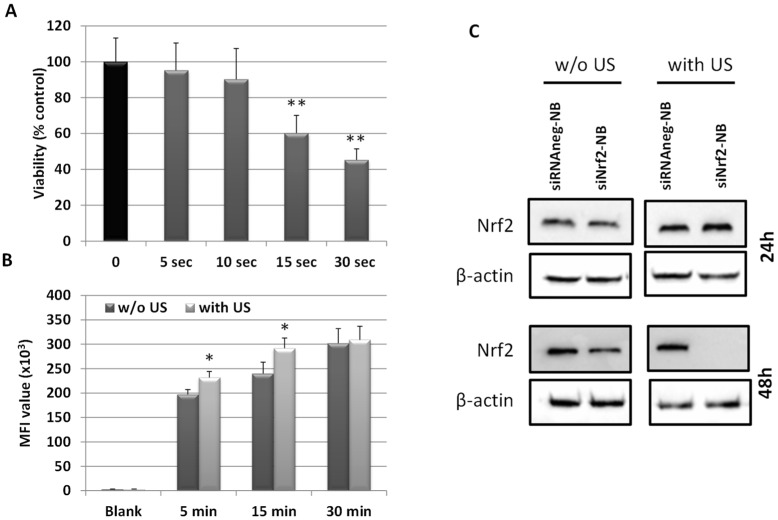
(**A**) Viability was determined by the MTT assay at 24 h in M14 cells exposed to US at the indicated times. Results are expressed as the percent of the relative control values and are the mean ± SD. ** *p* ≤ 0.01 vs. 0 s. (**B**) Fluorescent images of 6-coumarin-NB uptake in exposed (with US) or nonexposed (w/o US) M14 cells to US at the indicated time. Green fluorescence of 6-coumarin was examined by a cytofluorimetric analysis. Results are expressed as the median fluorescence intensity (MFI) values (means ± SD). * *p* ≤ 0.05 ** *p* ≤ 0.01 US exposed vs. unexposed US cells. (**C**) Western blot analysis of Nrf2 in M14 cells treated with siNrf2-NB or siRNAneg-NB in exposed (with US) or nonexposed (w/o US) to US at 24 h and 48 h.

First, we identified nontoxic insonation conditions in M14 cells. The cells were exposed to US with an oscillation frequency of 2.5 ± 0.1 MHz for 0, 5, 10, 15, 30, or 60 s. After 24 h, we observed a significant viability inhibition in cells insonated for 15, 30, and 60 s but not after 5 or 10 s of US exposure (Figure 6A). Thus, the following experiments were performed with a 10-s US exposure.

The cellular internalization of fluorescent 6-coumarin-NBs in M14 cells exposed to US was evaluated by a cytofluorimetric analysis. As shown in Figure 6B, a higher level of fluorescence was observed at 5 and 15 min in insonated M14 cells with respect to non-sonified cells. At 30 min, the fluorescence was similar in the two experimental groups. 

The transfection efficiency of siNrf2 was consistent with the NB uptake results. Indeed, siNrf2-NB inhibited Nrf2 protein expression at 48 h in non-sonified cells with respect to siRNAneg-NB-treated cells; however, in M14 exposed to US, the inhibition was complete (Figure 6C). Of note, we obtained a significant inhibition at 48 h but not at 24 h, unlike it is shown in Figure 4. Nevertheless, it is important to point out that the two experimental conditions were very different (see Section 2.10 and Section 2.14); therefore, the times were not comparable.

### 3.5. Echogenic Properties of NB Formulations

**Figure 7:**
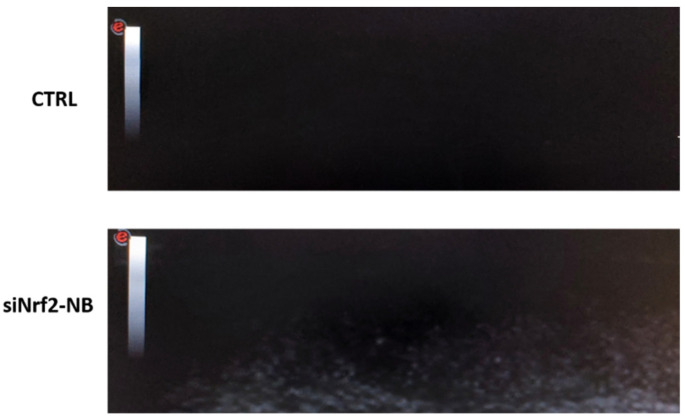
Representative snapshots of the B mode cineloops of the control experiment (ultrapure water without NBs) and siNrf2-NB (MI = 0.8, frequency = 12 MHz).

In addition to improving the transfection efficiency, polymer-shelled NBs combined with US can represent an advantageous tool for siRNA delivery, because their biodistribution can be detected using US imaging. As a consequence, NBs can be considered a theranostic system for both diagnosis and therapy. The possibility of US imaging siNrf2-NB visualization was investigated using a US clinical echomachine. The ultrasound experiments showed that siNrf2-NB had the ability to generate an echogenic response (Figure 7).

The NB decafluopentane core conferred them acoustically active properties. Indeed, US can trigger decafluoropentane liquid-to-vapor transition by means of the acoustic droplet vaporization (ADV) phenomenon, converting nanodroplets into bubbles [67,68]. 

## 4. Conclusions

The formulation strategy developed in this study allowed stable siRNA-loaded NBs to be obtained for the intracellular delivery of siRNA against Nrf2. The results support the role of Nrf2 in maintaining the chemoresistance in melanoma cells and suggest that its inhibition through siRNA could be a valuable strategy to overcome drug resistance. Moreover, NBs turn out to be an excellent siRNA delivery tool, enhanced by the use of US, eliciting a future possible clinical translation.

## Figures and Tables

**Table 1:** Physicochemical parameters of the siRNA-loaded NBs.

Formulation	Average Diameter ± SD (nm)	Polydispersity Index (PDI) ± SD	Zeta Potential ± SD (mV)	Osmolarity (mOsm)
NBs	102.3 ± 2.3	0.221 ± 0.02	27.5 ± 2.5	280
Fluorescent NBs	98.4 ± 5.0	0.220 ± 0.03	26.8 ± 2.2	282
siNrf2-NBs	97.5 ± 4.6	0.218 ± 0.02	26.2 ± 1.8	280
siRNAneg-NBs	100.2 ± 3.1	0.222 ± 0.01	26.4 ± 2.1	278

## Data Availability

The data presented in this study are available on request from the corresponding author.
